# P-985. Understanding the Effect of Frailty on People Living with HIV- Online CME Improves Knowledge and Confidence

**DOI:** 10.1093/ofid/ofae631.1175

**Published:** 2025-01-29

**Authors:** Sara Thorpe, Maria B Uravich, Emily Mikhael

**Affiliations:** Medscape Education, New York, New York; Medscape Education, New York, New York; Medscape, LLC, Savage, Minnesota

## Abstract

**Background:**

Frailty affects the clinical status of older people living with HIV (PLWH), increasing their risk of adverse outcomes while impacting quality of life and health span. This study evaluated the effect of an online CME curriculum on enhancing awareness and knowledge of contributing factors associated with frailty in PLWH, as well as improving confidence in supporting best practices for screening, management, and guidelines-based care for frail PLWH.

**Methods:**

The CME intervention comprised of a 30- minute online video-based panel discussion between 2 expert faculty. Response to 3 multiple choice, knowledge questions 1 self-efficacy, 5-point Likert scale confidence question were analyzed using a repeated pairs pre-/post-assessment study design. Pre- to post responses were compared using a McNemar’s test to assess statistical significance. The activity posted on 12/8/2023; data were collected through 3/7/2024.

**Results:**

The analysis set consisted of responses of HIV specialists (n=32) and Primary Care Physicians (n=244) who answered all pre/post questions. Analysis demonstrated a significant improvement in knowledge and confidence.

- 50% of HIV specialists (P< .001, 35% v. 52% pre to post) and 37% of primary care physicians (P< .0010, 29% v. 39% pre to post) demonstrated improvement across all learning objectives
- 57% relative increase among HIV specialists (P< .01, 44% v. 69% pre to post) and 47% relative increase among primary care physicians (P< .001, 34% v. 50% pre to post) in knowledge regarding the consequences of frailty in PLWH
- 42% relative increase among HIV specialists (P< .05, 31% v. 44% pre to post) and 22% relative increase among primary care physicians (P< .001 27% v. 33% pre to post) in knowledge regarding screening tools for frailty assessment
- 63% improved confidence in the use of available tools to screen/assess frailty in PLWH aged 50+

**Conclusion:**

This study demonstrated the success of online, video-based panel discussion CME on improving knowledge and confidence related to individualizing and improving care aging populations who are living with HIV. These findings suggest the benefits of education that addresses clinicians’ individual needs across the continuum of their professional development.

**Disclosures:**

**All Authors**: No reported disclosures

